# Supplementary material for: Effects of Coleus amboinicus L. Essential Oil and Ethanolic Extracts on Planktonic Cells and Biofilm Formation of Microsporum canis Isolated from Feline Dermatophytosis
Source: Antibiotics (Basel). 2022 Dec 1;11(12):1734. doi: 10.3390/antibiotics11121734 (PMC9774219; doi:10.3390/antibiotics11121734)
Supplement: Supplementary file 1 [file antibiotics-11-01734-s001.zip › Antibiotics-2026194 27-11-22 Supplementary 1-Mass spectra.pdf]

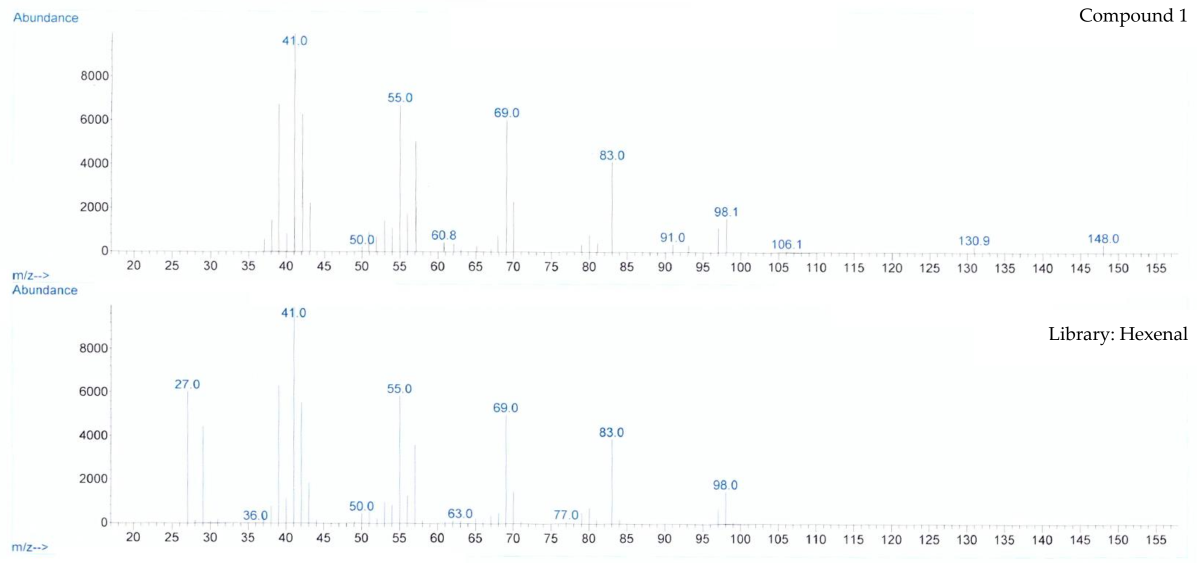
Compound 1
Abundance
41.0
55.0
69.0
83.0
50.0
60.8
91.0
98.1
106.1
130.9
148.0
m/z-->
Library: Hexenal
Abundance
41.0
27.0
55.0
69.0
83.0
36.0
50.0
63.0
77.0
98.0
m/z-->

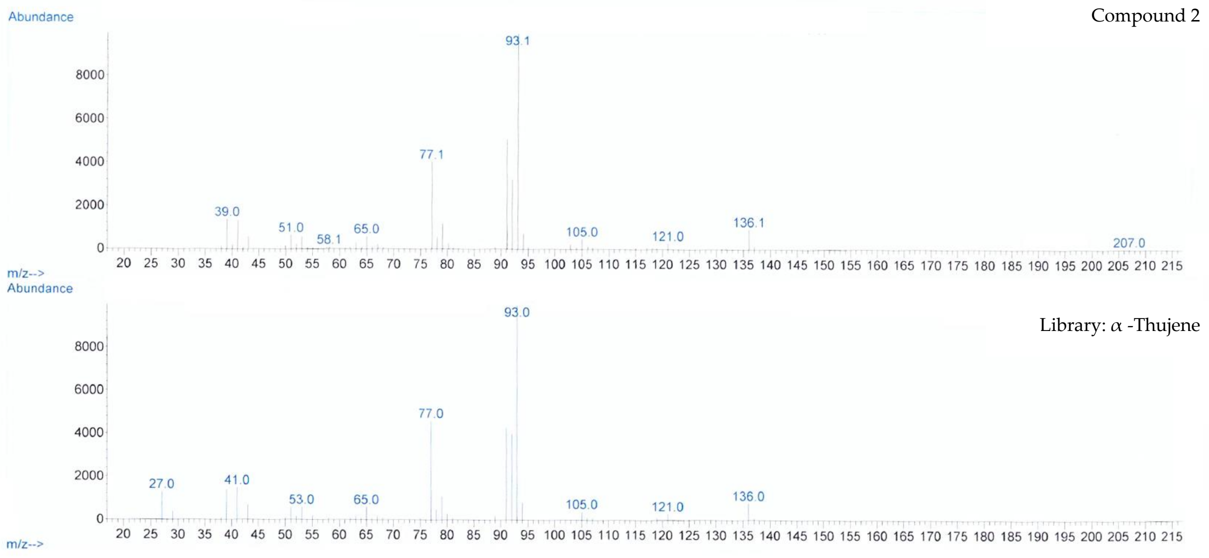
Compound 2
Abundance
93.1
77.1
39.0
51.0
58.1
65.0
105.0
121.0
136.1
207.0
m/z-->
Library: α -Thujene
Abundance
93.0
77.0
27.0
41.0
53.0
65.0
105.0
121.0
136.0
m/z-->

Compound 3

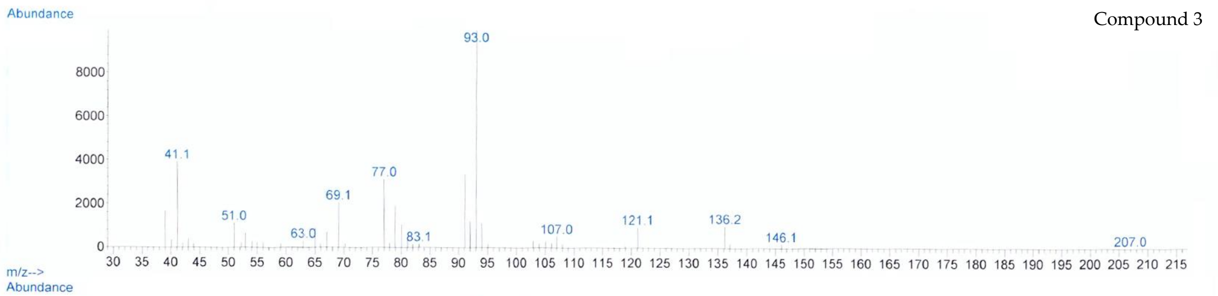

Library: Sabinene

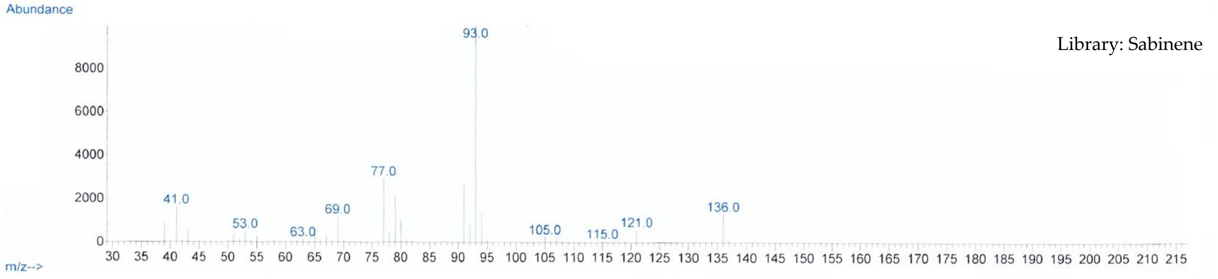

Compound 4

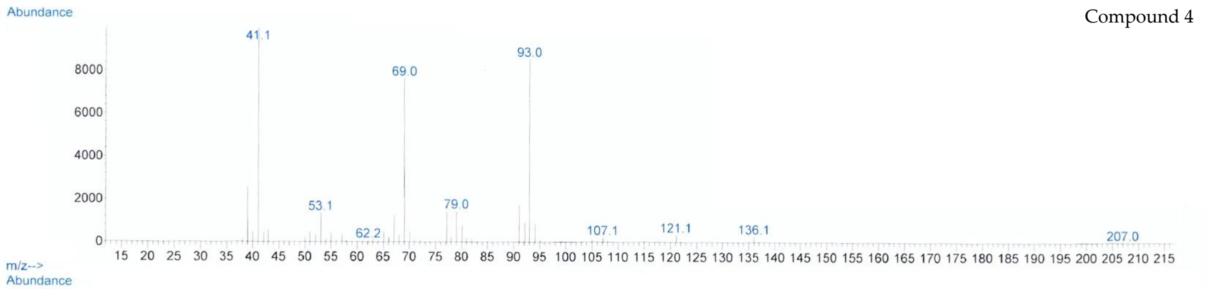

Library: β -Myrcene

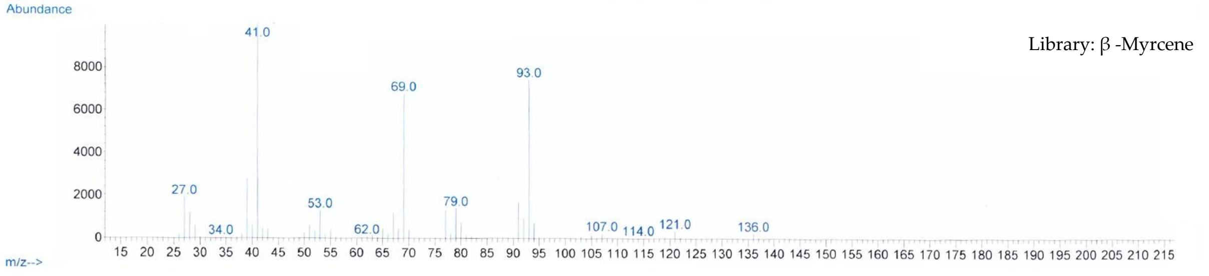

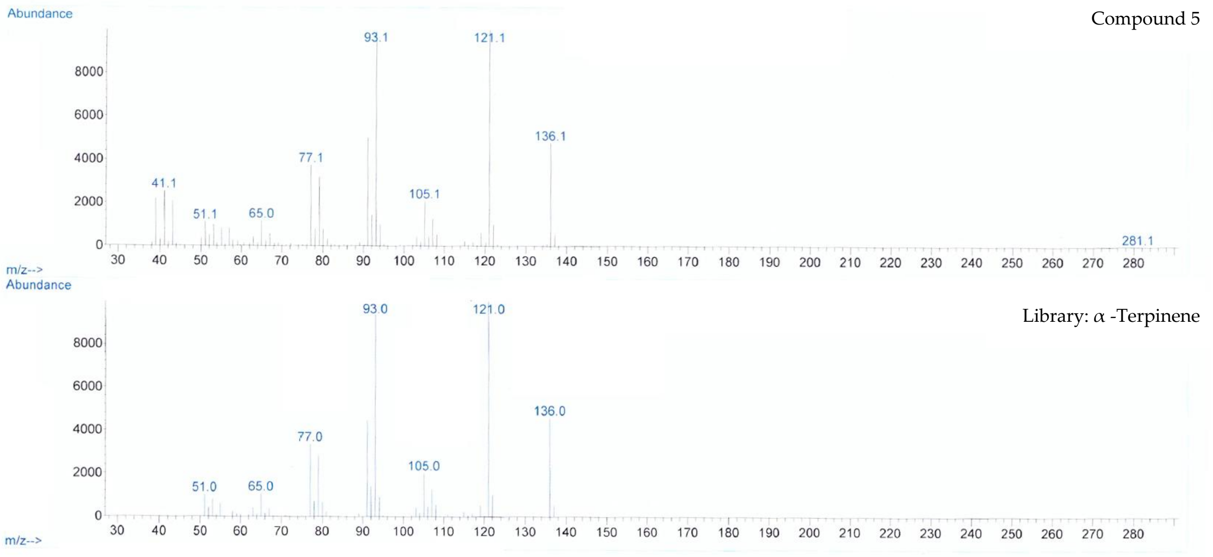
Abundance
Compound 5
93.1
121.1
136.1
77.1
41.1
105.1
51.1
65.0
281.1
8000
6000
4000
2000
0
m/z-->
30 40 50 60 70 80 90 100 110 120 130 140 150 160 170 180 190 200 210 220 230 240 250 260 270 280
Abundance
Library: α -Terpinene
93.0
121.0
136.0
77.0
105.0
51.0
65.0
8000
6000
4000
2000
0
m/z-->
30 40 50 60 70 80 90 100 110 120 130 140 150 160 170 180 190 200 210 220 230 240 250 260 270 280

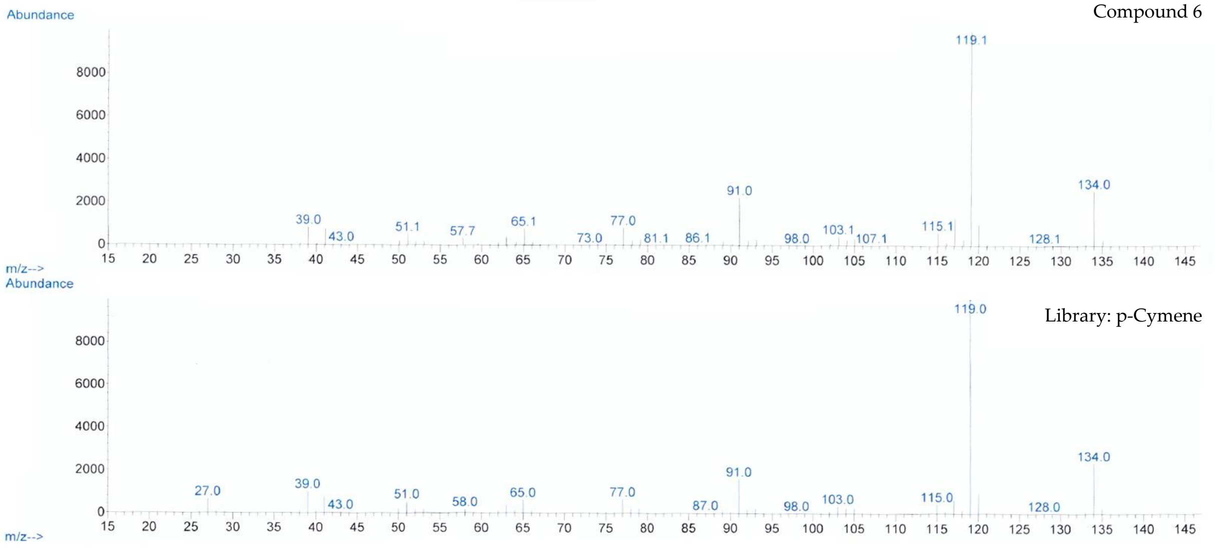
Abundance
Compound 6
119.1
134.0
91.0
39.0
43.0
51.1
57.7
65.1
73.0
77.0
81.1
86.1
98.0
103.1
107.1
115.1
128.1
8000
6000
4000
2000
0
m/z-->
15 20 25 30 35 40 45 50 55 60 65 70 75 80 85 90 95 100 105 110 115 120 125 130 135 140 145
Abundance
Library: p-Cymene
119.0
134.0
91.0
27.0
39.0
43.0
51.0
58.0
65.0
77.0
87.0
98.0
103.0
115.0
128.0
8000
6000
4000
2000
0
m/z-->
15 20 25 30 35 40 45 50 55 60 65 70 75 80 85 90 95 100 105 110 115 120 125 130 135 140 145

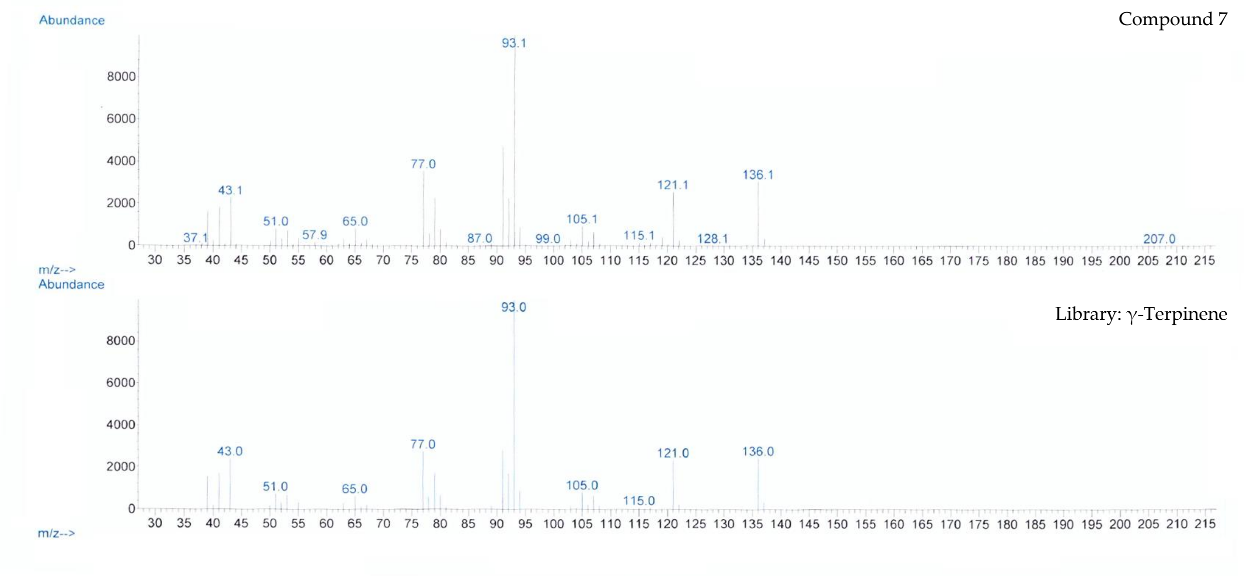
Compound 7
Library: γ-Terpinene
Abundance
m/z-->
93.1
77.0
43.1
121.1
136.1
37.1
51.0
57.9
65.0
87.0
99.0
105.1
115.1
128.1
207.0
93.0
43.0
77.0
121.0
136.0
51.0
65.0
105.0
115.0

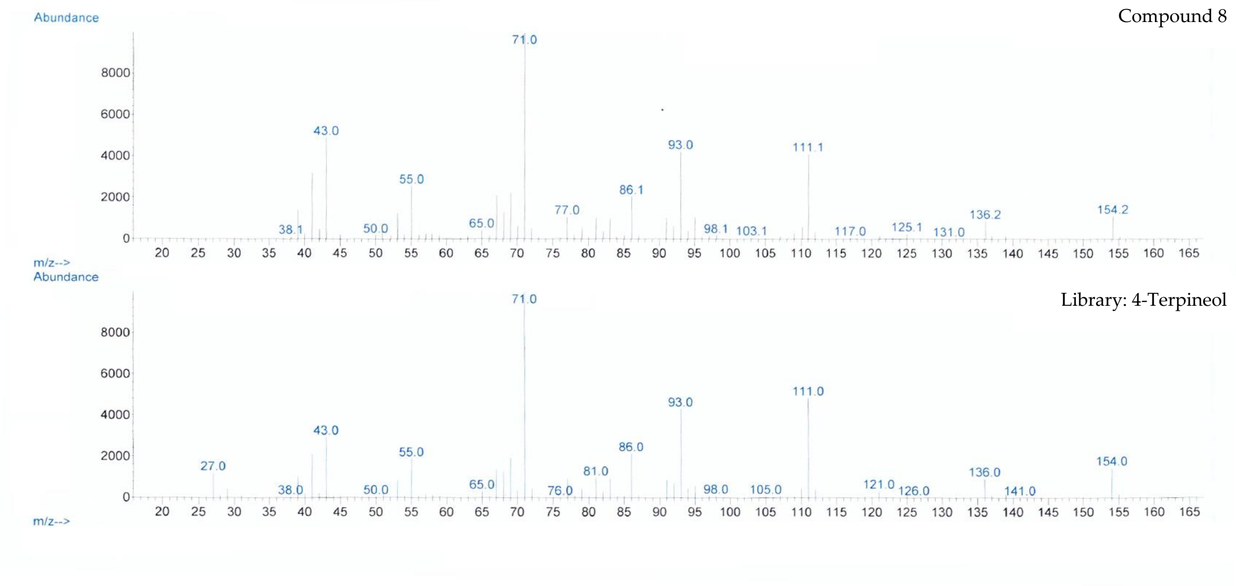
Compound 8
Library: 4-Terpineol
Abundance
m/z-->
71.0
43.0
93.0
111.1
55.0
86.1
77.0
136.2
154.2
38.1
50.0
65.0
98.1
103.1
117.0
125.1
131.0
71.0
93.0
111.0
43.0
55.0
86.0
27.0
81.0
136.0
154.0
38.0
50.0
65.0
76.0
98.0
105.0
121.0
126.0
141.0

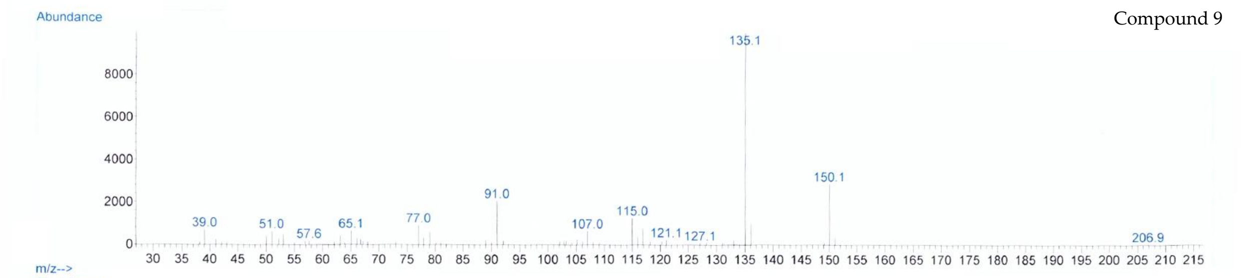
Compound 9
Abundance
8000
6000
4000
2000
0
m/z-->
39.0
51.0
57.6
65.1
77.0
91.0
107.0
115.0
121.1
127.1
135.1
150.1
206.9
30 35 40 45 50 55 60 65 70 75 80 85 90 95 100 105 110 115 120 125 130 135 140 145 150 155 160 165 170 175 180 185 190 195 200 205 210 215

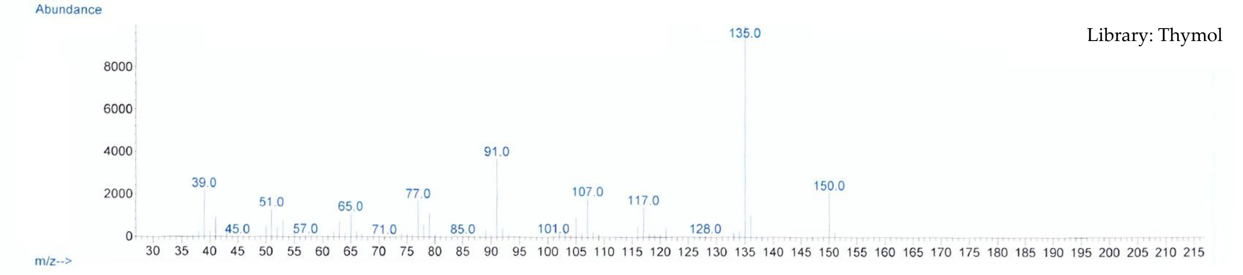
Library: Thymol
Abundance
8000
6000
4000
2000
0
m/z-->
39.0
45.0
51.0
57.0
65.0
71.0
77.0
85.0
91.0
101.0
107.0
117.0
128.0
135.0
150.0
30 35 40 45 50 55 60 65 70 75 80 85 90 95 100 105 110 115 120 125 130 135 140 145 150 155 160 165 170 175 180 185 190 195 200 205 210 215

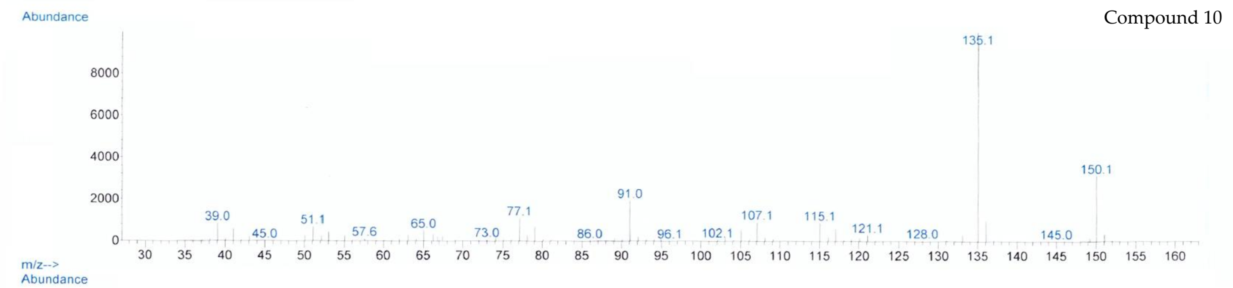
Compound 10
Abundance
8000
6000
4000
2000
0
m/z-->
39.0
45.0
51.1
57.6
65.0
73.0
77.1
86.0
91.0
96.1
102.1
107.1
115.1
121.1
128.0
135.1
145.0
150.1
30 35 40 45 50 55 60 65 70 75 80 85 90 95 100 105 110 115 120 125 130 135 140 145 150 155 160

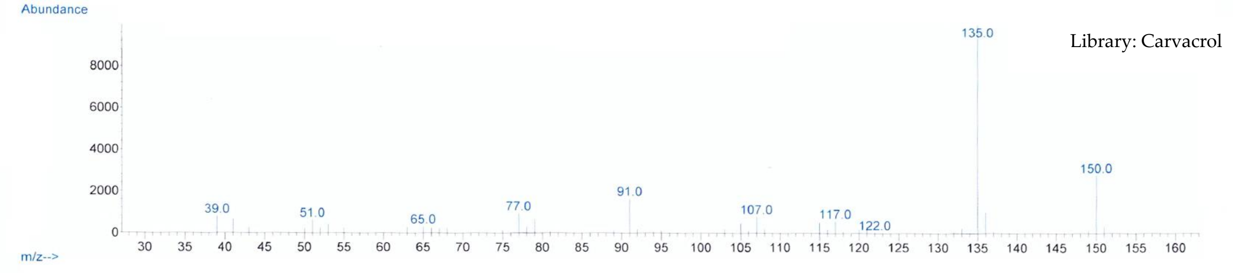
Library: Carvacrol
Abundance
8000
6000
4000
2000
0
m/z-->
39.0
51.0
65.0
77.0
91.0
107.0
117.0
122.0
135.0
150.0
30 35 40 45 50 55 60 65 70 75 80 85 90 95 100 105 110 115 120 125 130 135 140 145 150 155 160

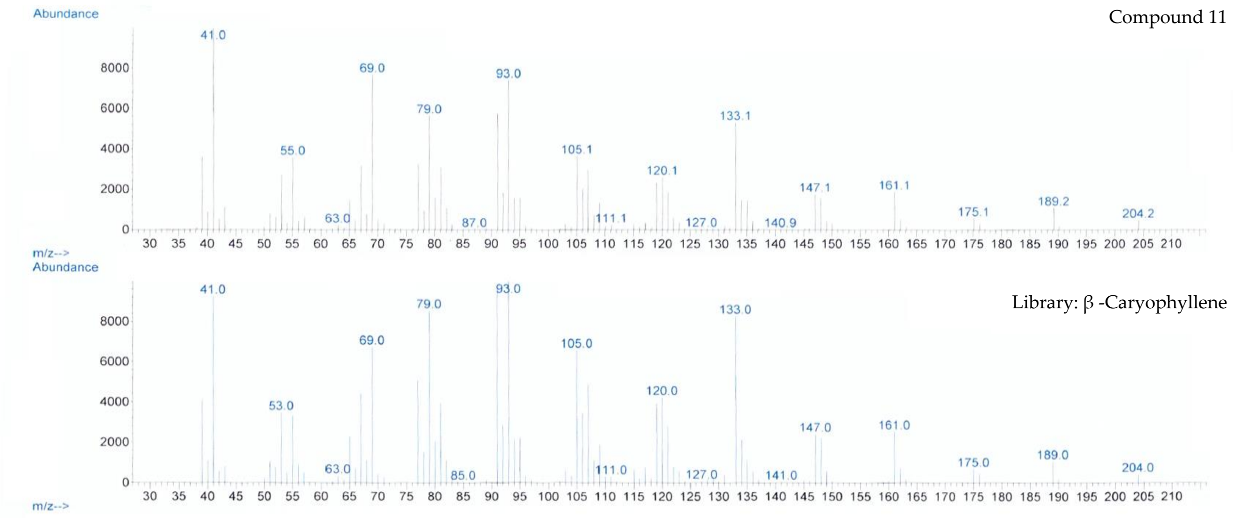
Compound 11
Library: β -Caryophyllene
Abundance
m/z-->
41.0
69.0
93.0
79.0
133.1
105.1
55.0
120.1
147.1
161.1
175.1
189.2
204.2
63.0
87.0
111.1
127.0
140.9
53.0
133.0
105.0
120.0
147.0
161.0
175.0
189.0
204.0
85.0
111.0
141.0

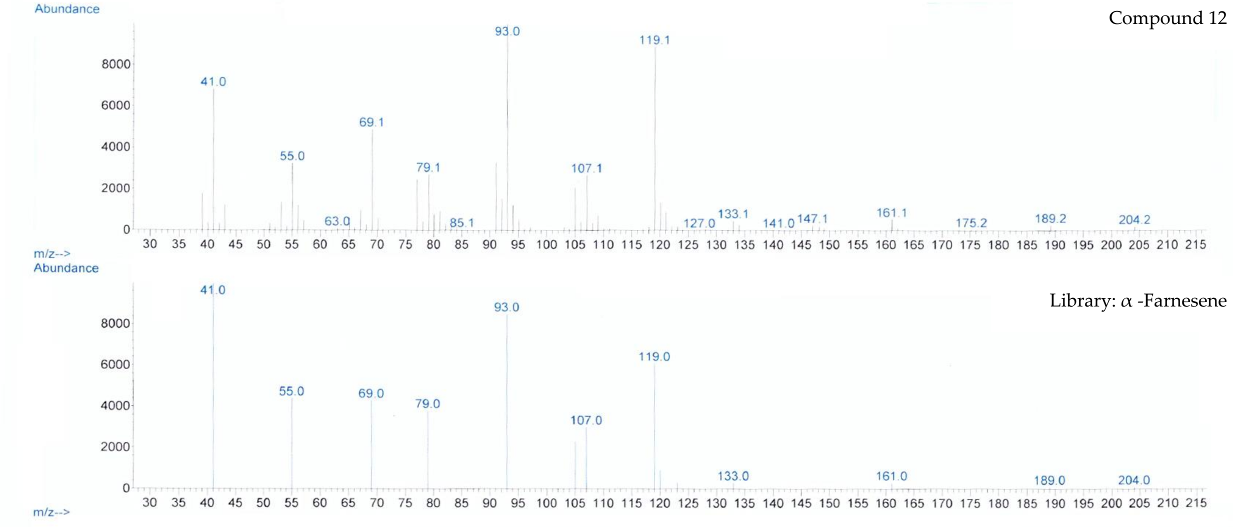
Compound 12
Library: α -Farnesene
Abundance
m/z-->
93.0
119.1
41.0
69.1
55.0
79.1
107.1
63.0
85.1
127.0
133.1
141.0
147.1
161.1
175.2
189.2
204.2
119.0
55.0
69.0
79.0
107.0
133.0
161.0
189.0
204.0

Compound 13

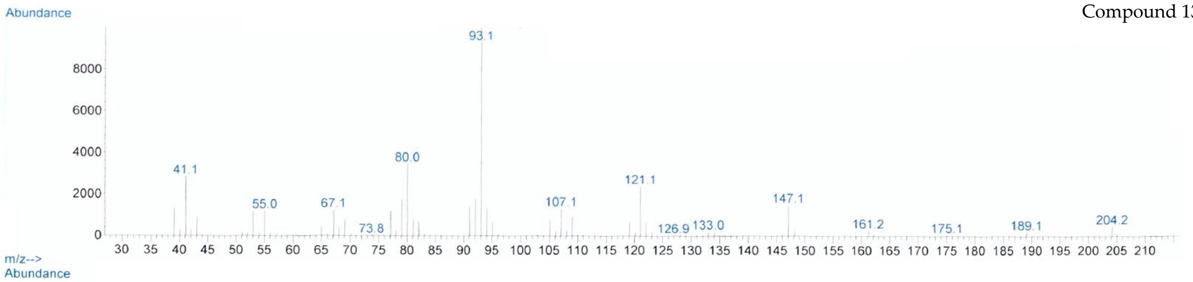

Library: α -Humulene

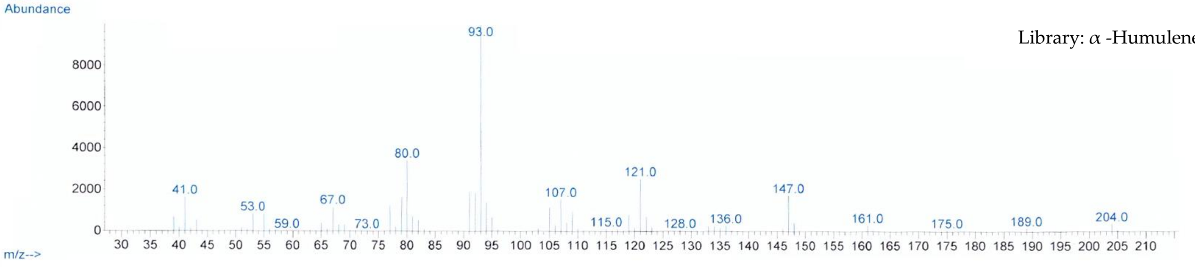

Compound 14

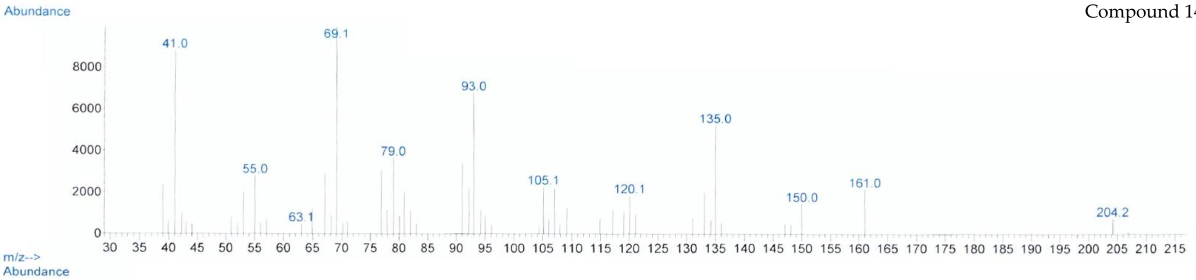

Library: β -Farnesene

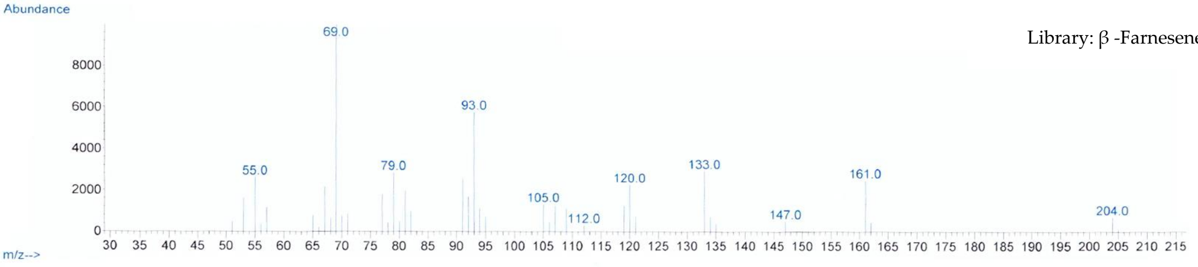

Compound 15

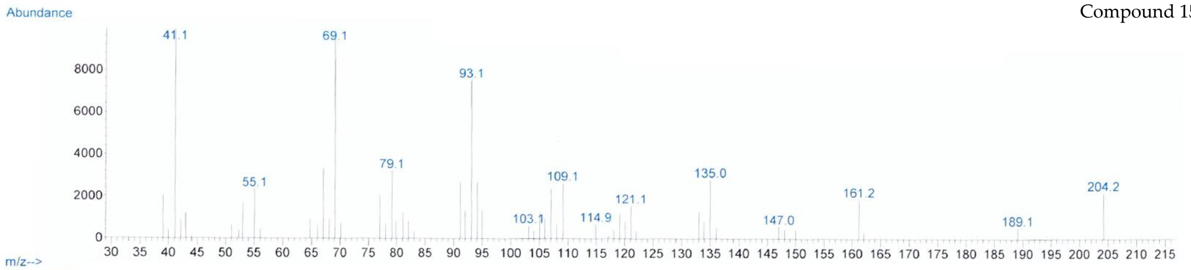

Library: β -Bisabolene

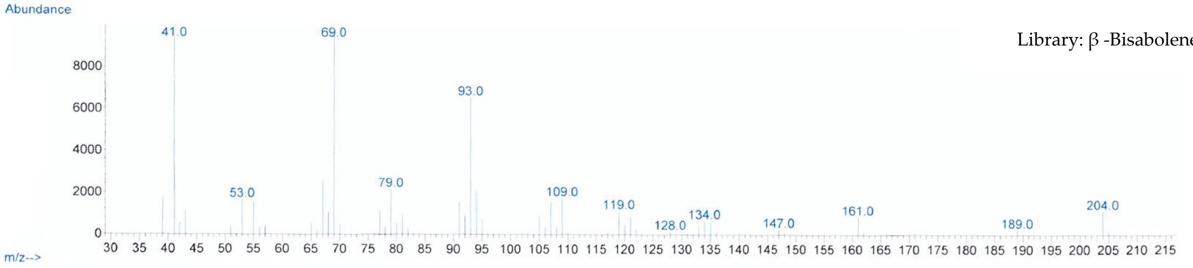

Compound 16

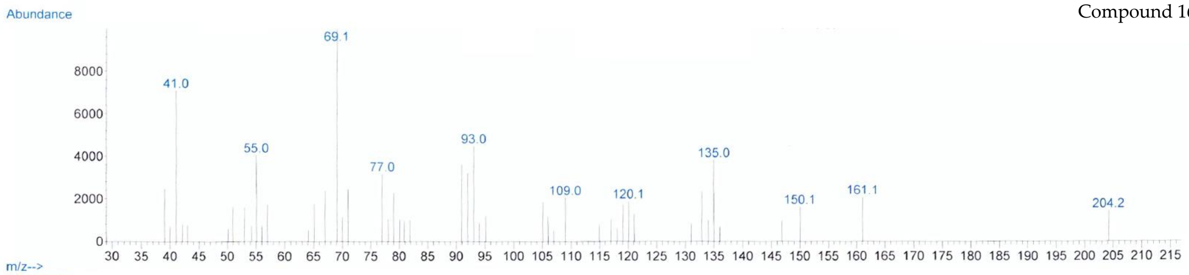

Library: β -Sesquiphellandrene

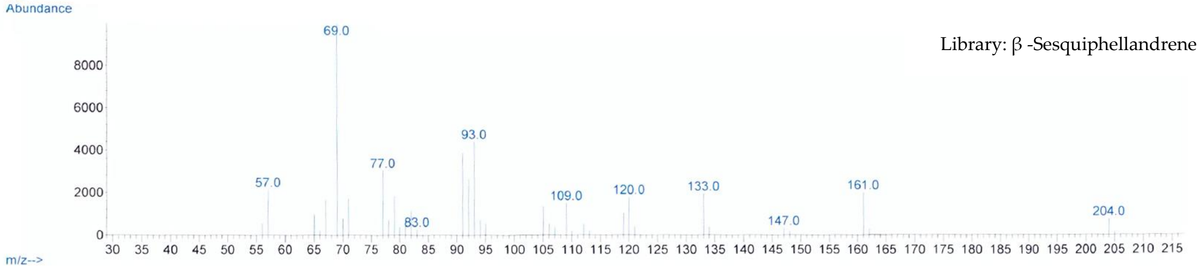

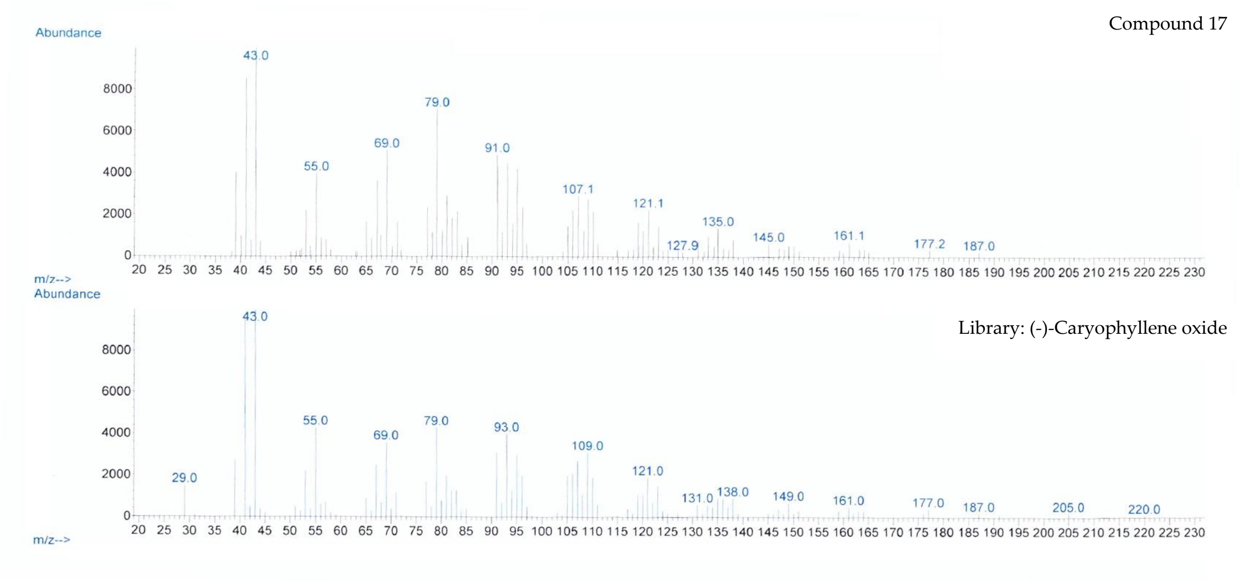

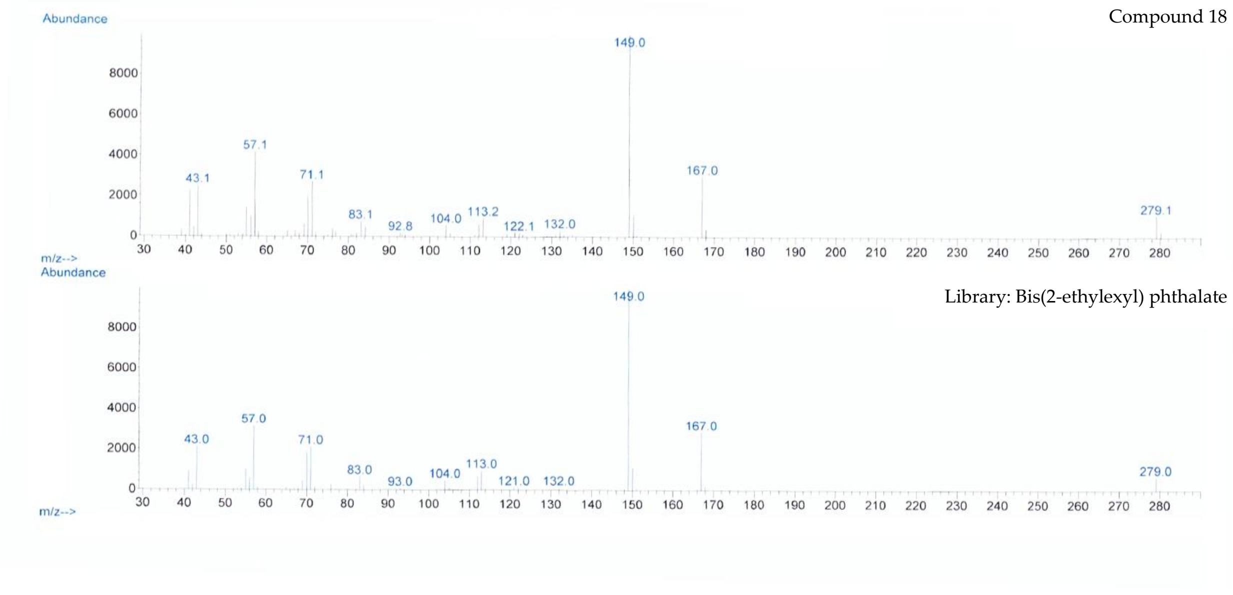

Figure S1: Mass spectrum of the main components of *Coleus amboinicus* essential oil determined via gas chromatography–mass spectrometry
