# Supplementary material for: Effects of Coleus amboinicus L. Essential Oil and Ethanolic Extracts on Planktonic Cells and Biofilm Formation of Microsporum canis Isolated from Feline Dermatophytosis
Source: Antibiotics (Basel). 2022 Dec 1;11(12):1734. doi: 10.3390/antibiotics11121734 (PMC9774219; doi:10.3390/antibiotics11121734)
Supplement: Supplementary file 1 [file antibiotics-11-01734-s001.zip › Antibiotics-2026194 27-11-22 Supplementary 2-Phylogenetic tree.pdf]

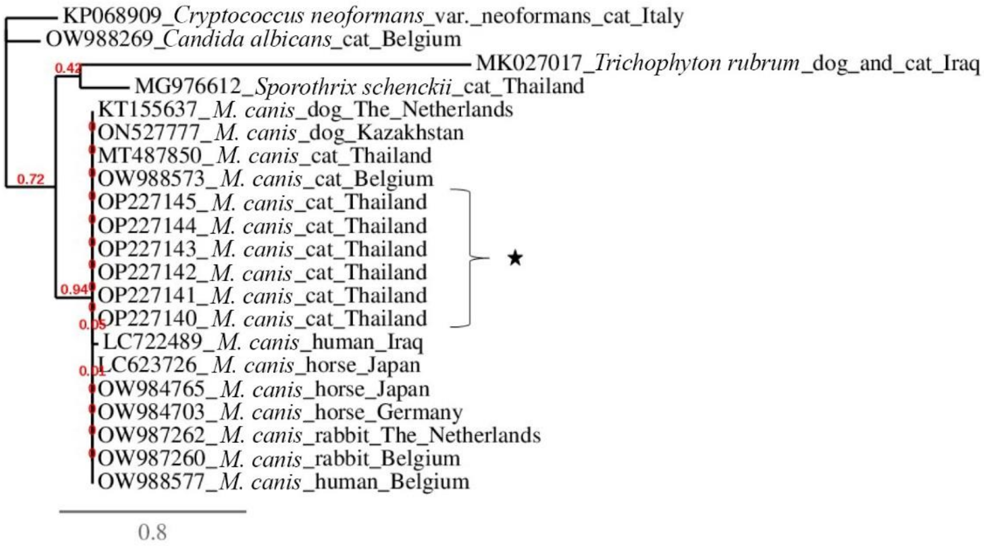

Figure S2: Phylogenetic tree analysis of *Microsporum canis* based on nucleotide sequences from a 720 bp fragment of ITS1-5.8S-ITS2 using the neighbor-joining method. Sequences from this study are marked with stars
