# Supplementary material for: Effects of Coleus amboinicus L. Essential Oil and Ethanolic Extracts on Planktonic Cells and Biofilm Formation of Microsporum canis Isolated from Feline Dermatophytosis
Source: Antibiotics (Basel). 2022 Dec 1;11(12):1734. doi: 10.3390/antibiotics11121734 (PMC9774219; doi:10.3390/antibiotics11121734)
Supplement: Supplementary file 1 [file antibiotics-11-01734-s001.zip › Antibiotics-2026194 27-11-22 Supplementary 3-Biofilm.pdf]

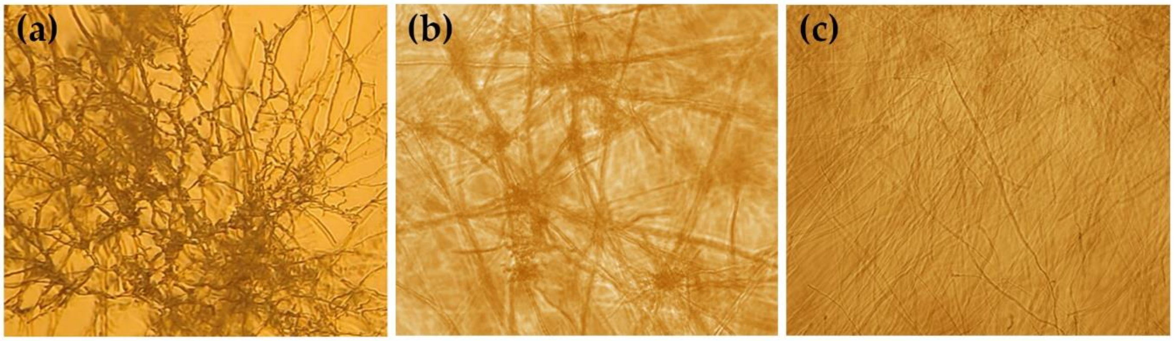

Figure S3: Microscopic appearance of *Microsporum canis* biofilm classified as strong (a), moderate (b), and weak (c) biofilm producers after 72 h incubation. (40× magnification).
